# Functional recovery from refractory hepatic encephalopathy following angiographic obliteration of a large, spontaneous portal-umbilical portosystemic shunt: a case report

**DOI:** 10.1186/s42155-022-00320-3

**Published:** 2022-08-24

**Authors:** Natalie L. Y. Ngu, Edward Saxby, Caitlin C. Farmer, Stuart Lyon, Suong Le

**Affiliations:** 1 Department of Gastroenterology and Hepatology, Monash Health, Level 3, 246 Clayton Rd, Clayton, VIC 3168 Australia; 2 School of Medicine, Nursing and Health Sciences, Monash University, Melbourne, Australia; 3 Monash Imaging, Monash Health, Clayton, Australia; 4 Western Health Medical Imaging, Western Health, Footscray, Australia

**Keywords:** Portal hypertension, Portosystemic shunt, Hepatic encephalopathy, Retrograde transvenous obliteration, Angiography

## Abstract

**Background:**

Hepatic encephalopathy (HE) as a consequence of cirrhosis with portal hypertension has a profound impact on quality of life for both patients and caregivers, has no gold-standard diagnostic test, and is a risk factor for mortality. Spontaneous portosystemic shunts (SPSS) are common in patients with cirrhosis, can be challenging to identify, and in some cases, can drive refractory HE. Cross-sectional shunt size greater than 83mm^2^ is associated with liver disease severity, overt HE, and mortality.

**Case presentation:**

We report a patient with refractory HE and frequent hospitalization in the context of an occult spontaneous portal-umbilical portosystemic shunt with an estimated cross-sectional area of 809mm^2^. Following identification and angiographic retrograde transvenous obliteration of the SPSS using plugs, coils and sclerosant, there was improvement in neurocognitive testing and no further hospitalization for HE.

**Conclusion:**

SPSS in the context of cirrhosis with portal hypertension can contribute to the debilitating effects of refractory HE. This case highlights the opportunity to search for SPSS in patients with HE unresponsive to therapy as angiographic obliteration is usually safe, well-tolerated, and clinically effective.

## Background

Hepatic encephalopathy (HE) is defined as “brain dysfunction caused by liver insufficiency and/or portosystemic shunts”, occurring in up to 30–45% with cirrhosis (American 2014). It can have a profound impact on quality of life for both patients and caregivers, has no gold-standard diagnostic test, and is a risk factor for mortality (D'Amico et al. 1986). Spontaneous portosystemic shunts (SPSS) are a common consequence of portal hypertension, with increasing size correlating with liver disease severity, risk of HE, and mortality (Simón-Talero et al. 2018).

Angiographic retrograde transvenous obliteration (ARTO), with or without balloon-occlusion has been developed for treatment of both gastric varices (Kumamoto et al. 2010)and refractory HE (Mukund et al. 2012)by impeding collateral flow through shunts. Alternative methods of venous obliteration include plugs, coils and sclerosing agents. ARTO for refractory HE has found to be safe and effective for at least 4–12 months in patients with cirrhosis failing medical management (Mukund et al. 2012; Lynn et al. 2016).

## Case presentation

**Fig. 1:**
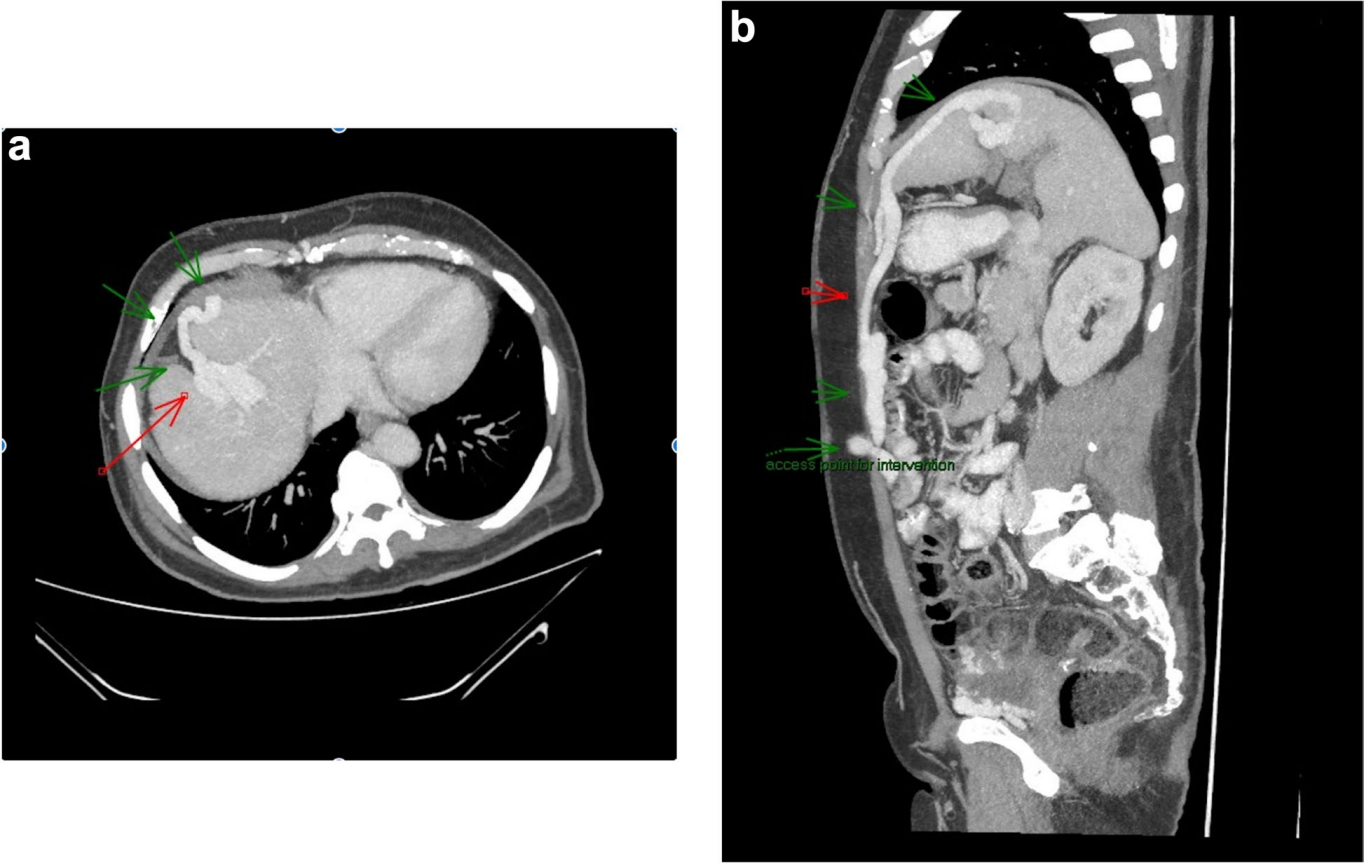
**a** Axial views of SPSS origin and upper abdominal trajectory. **b** Sagittal views of SPSS origin and upper abdominal trajectory

**Fig. 2:**
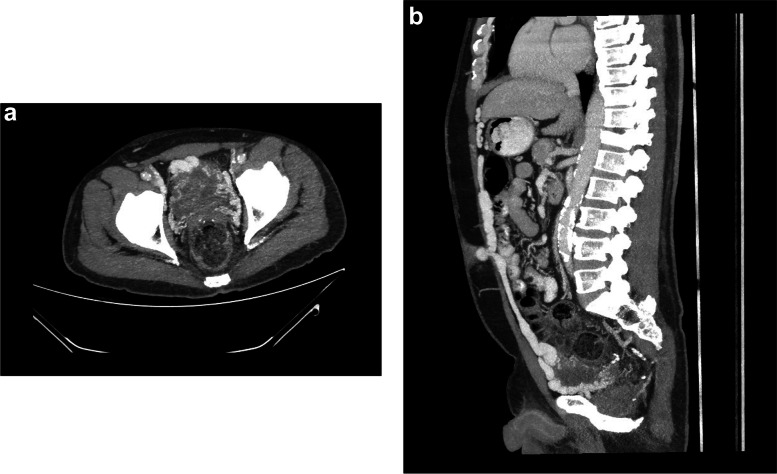
**a** Axial views of inferior SPSS. **b** Sagittal views of inferior SPSS

**Fig. 3:**
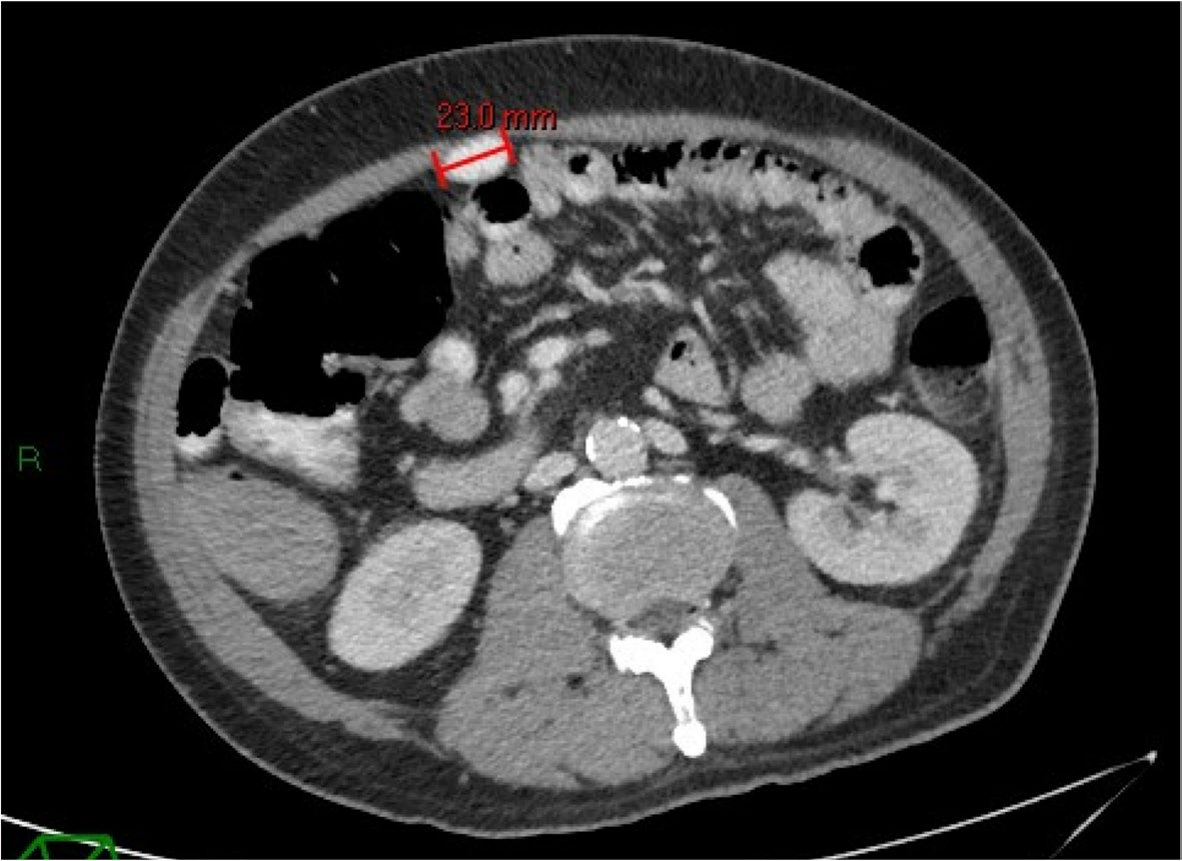
Portoumbilical shunt widest diameter assessment = 23 mm

**Fig. 4:**
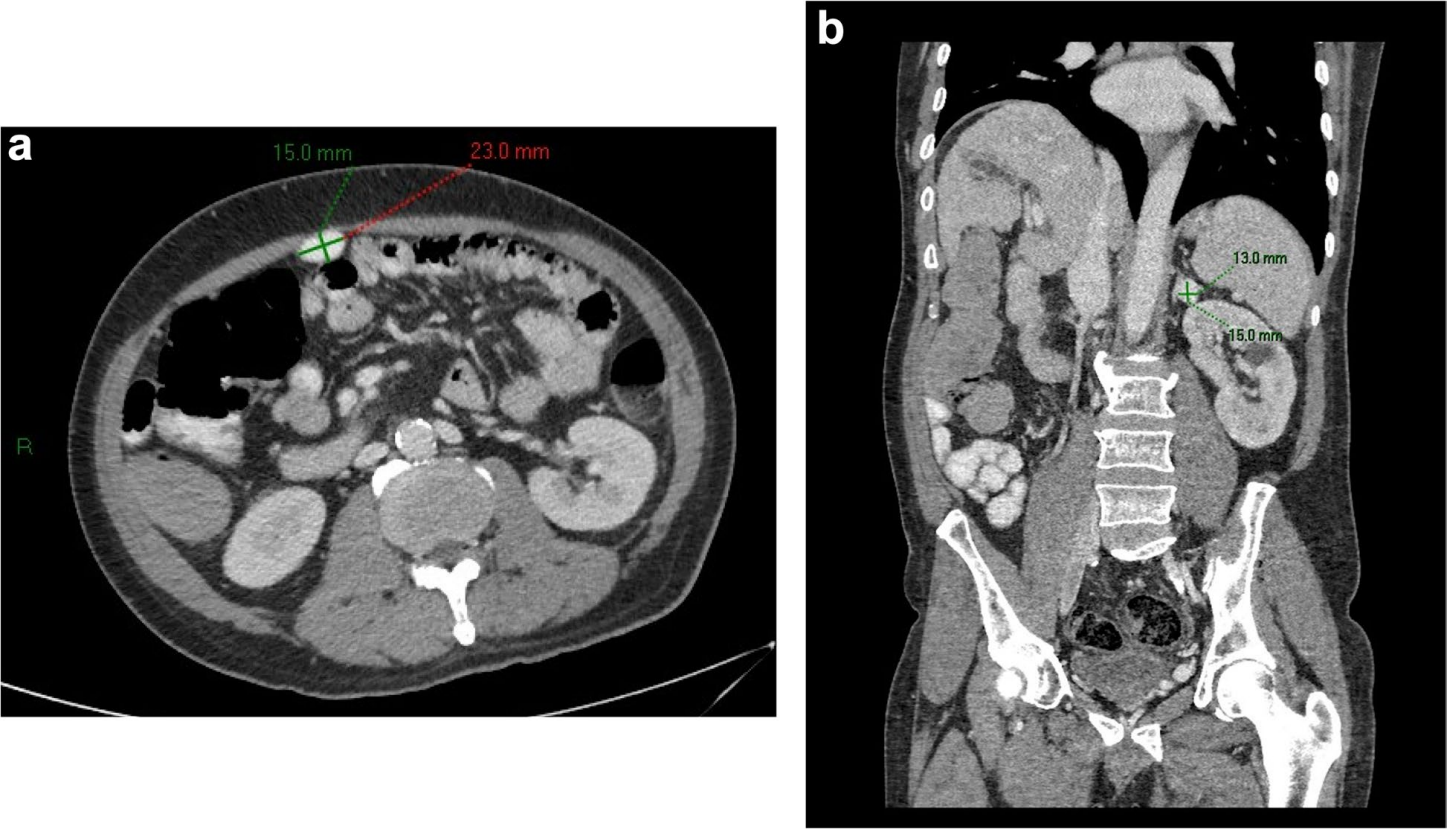
**a** and **b** Representative images of shunt diameter measurement technique

A 53-year-old man with cirrhosis and ongoing alcohol intake excess presented to our tertiary hospital with decreased conscious state, clinically consistent with Westhaven Grade 2–3 HE, which improved but did not resolve following treatment with lactulose, rifaximin and nutritional supplementation. Investigations for other causes for altered conscious state, including brain computed tomography (CT), blood glucose levels, and biochemistry, were unremarkable, and there was no history of sedating medication use or active infection. Investigations for an underlying precipitant included abdominal CT, which demonstrated extensive anterior abdominal wall varices including recanalization and prominence of the umbilical vein. The patient had three further admissions for HE over the next four months, was unable to work, and his partner experienced significant carer stress. In consultation with an experienced interventional radiologist, the large portal-umbilical shunt was identified as a potential cause for hepatic encephalopathy (Fig. 1a and b: axial and sagittal views of SPSS origin and upper abdominal trajectory). This vessel can be seen draining inferiorly into engorged pelvic veins (Fig. 2a and b: axial and sagittal views of inferior SPSS). The SPSS was 23 mm at widest diameter (Fig. 3: portoumbilical shunt widest diameter assessment = 23 mm), with a total cross-sectional area of 809 mm^2^using Baveno VI-SPSS Group criteria (Praktiknjo et al. 2020), calculated by adding the maximal cross-sectional areas of all portosystemic shunt vessels greater than 6 mm in diameter, calculated on the basis of  r^2^ (noting accompanying, less prominent splenorenal and gastric varices); (Fig. 4a and b: representative images of shunt diameter measurement technique).

**Fig. 5:**
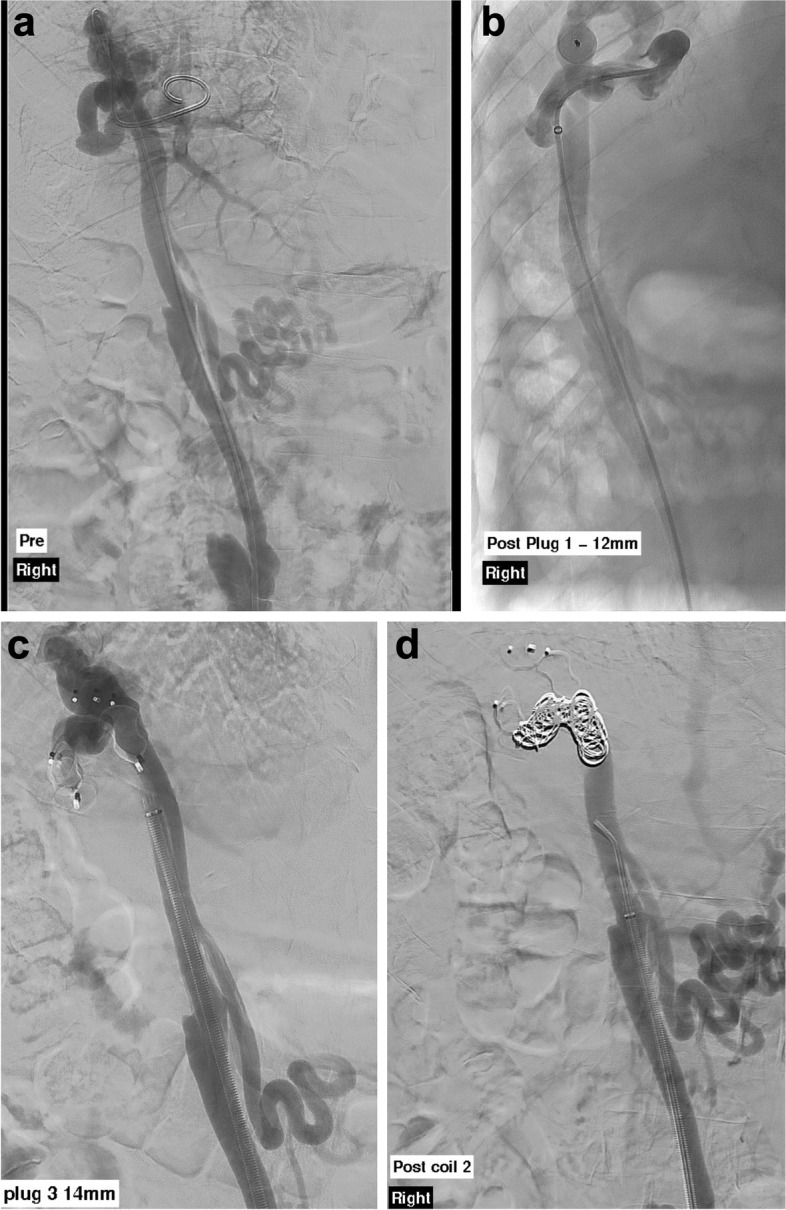
**a**-**d** Intraoperative images with sequential placement of Amplatzer plugs and pushable coils

**Fig. 6:**
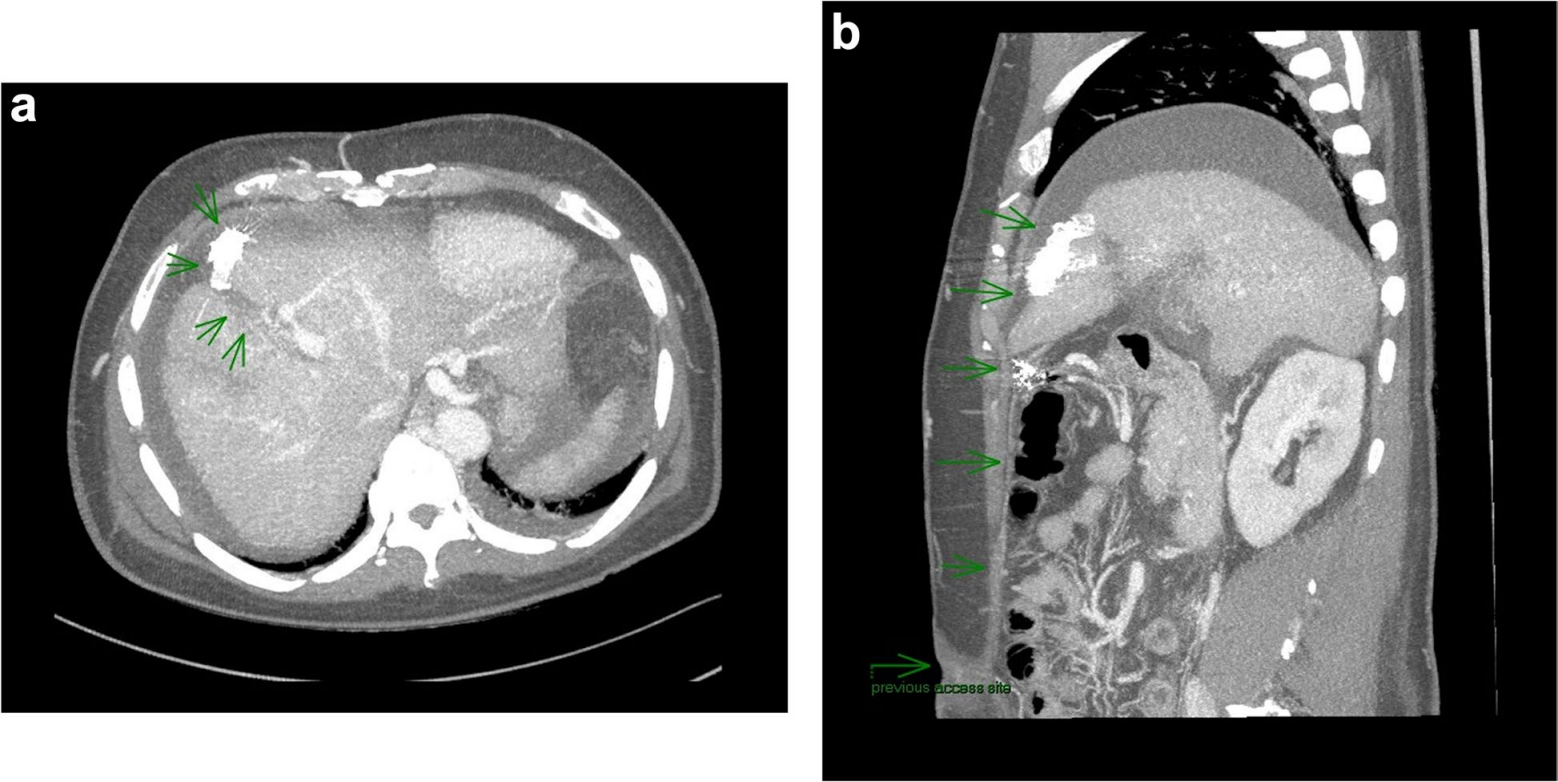
**a** Axial images demonstrating absence of the previously seen SPSS with embolization material in situ. **b** Sagittal images demonstrating absence of the previously seen SPSS with embolization material in situ

The patient subsequently underwent an ARTO to occlude the portosystemic shunt, allowing for increased hepatic filtration of portal blood via reduction of bypass, thereby reducing cerebrotoxic metabolite volume and associated encephalopathy. The umbilical vein was accessed under ultrasound guidance with retrograde introduction of an 8 French sheath, through which angiographic characterisation (Fig. 5: intraoperative images with sequential placement of Amplatzer plugs and pushable coils) and subsequently satisfactory occlusion of the portal-to-venous umbilical shunt was achieved, using 12 mm type 2 and 3, and 14 mm type 2 Amplatzer plugs (Abbott Medical, USA), 15 mm, 12 mm and 10 mm Interlock pushable coils (Boston Scientific, Ireland) and then Fibrovein to sclerose the umbilical vein, thereby reducing the chance of post procedure shunt recurrence. No retrograde flow into the portal venous system was elicited at the case conclusion. There were no periprocedural complications. Follow-up ultrasound at seven days confirmed successful SPSS occlusion. A further CT was performed at six weeks, confirming eradication of the target SPSS (Fig. 6a and b: axial and sagittal images demonstrating absence of the previously seen SPSS with embolization material in situ).

**Fig. 7:**
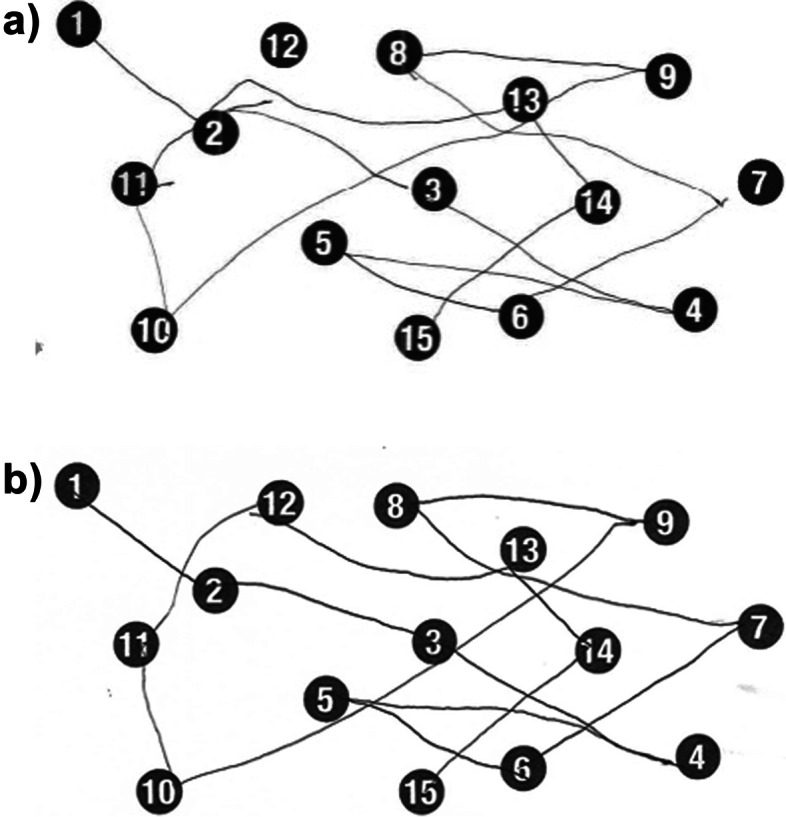
Number Connection Test **a**) Prior to ARTO (62 s to complete) and **b**) At 2 weeks from ARTO (taking 55 s to complete)

**Fig. 8:**
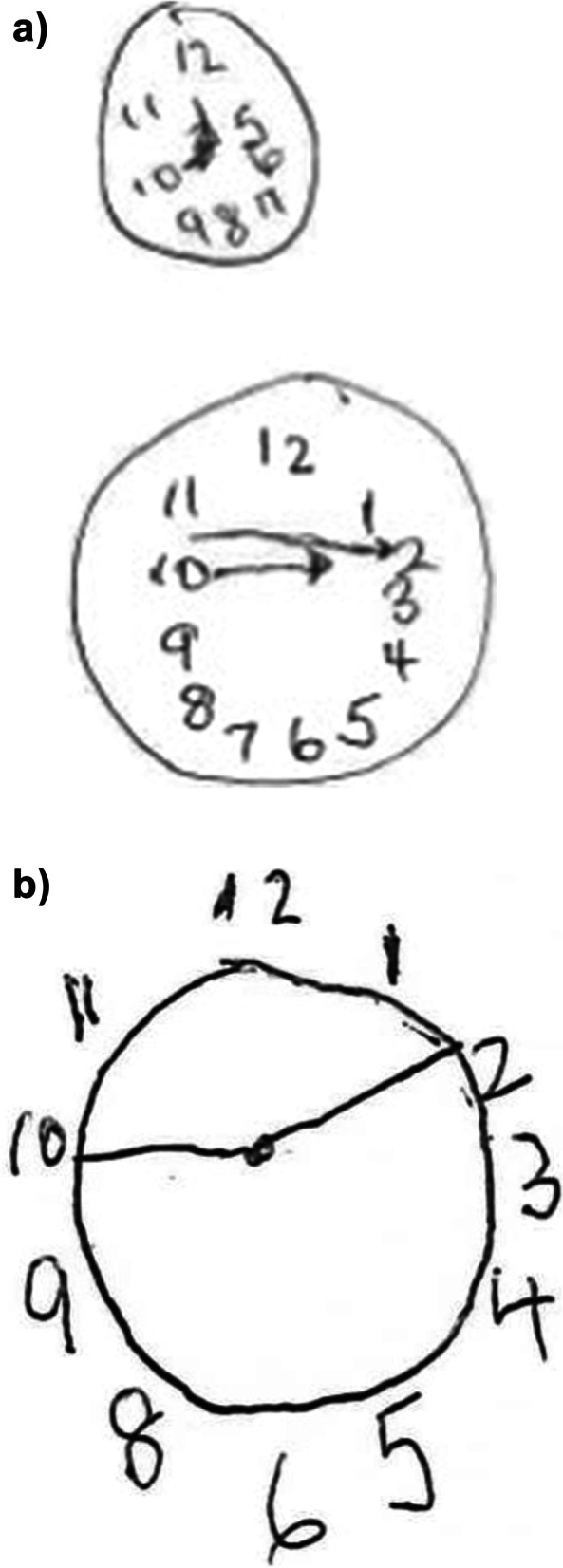
Clockface drawing with patient instructed to draw the hands at “10 to 2” **a**) Prior to ARTO and **b**) At 2 weeks from ARTO

Neurocognitive testing was conducted at baseline and at two weeks post-ARTO (Figs. 7 and 8). Baseline tests demonstrated impairment, including delayed time to completion of the Number Connection Test at 62 s and an improvement to 55 s at 2 week follow up. The baseline clockface drawing demonstrated incomplete numbers and inaccurate hand placement with subsequent improvement at 2 weeks. This improvement correlated with absence of emergency hospital admissions in the three months post-ARTO.

This case report did not require institutional ethics committee approval. Written, informed consent was obtained from the patient for publication of this case report and any accompanying media.

## Discussion

Our patient with refractory HE and a large, occult SPSS successfully underwent ARTO with benefits persisting both radiologically and clinically at six weeks. This case is unique in several aspects including a) the size of the SPSS at 10 × the threshold for risk of HE and death, b) initial delay in identification of the clinical significance of the SPSS to this patient’s presentations, and c) documented clinical and cognitive improvement following angiographic intervention.

At 809mm^2^, the SPSS identified in this case is far greater than the threshold for adverse events identified by Praktiknjo et al., who additionally found that a total surface area of greater than 83mm^2^was associated with higher model for end-stage liver disease score, history of overt HE, and lower 1-year survival compared to smaller SPSS (Praktiknjo et al. 2020). In addition to surface area, a diameter threshold of 8 mm (Simón-Talero et al. 2018) compared to 23 mm in our patient, as well as total number of SPSS, are both associated with presence of overt HE.

SPSS are present in 30–60% of those with cirrhosis (Simón-Talero et al. 2018). Common sites include gastrorenal, splenorenal, paraumbilical, esophageal SPSS and additional complications include variceal bleeding, portal vein thrombosis and deterioration in liver disease (Nardelli et al. 2020). Most SPSS can be visualized on CT, as demonstrated in a study of 222 patients with cirrhosis who underwent CT abdomen with portal venous phase contrast, with an SPSS identified in 63.5% by two experienced radiologists (Nardelli et al. 2021). As demonstrated in our case, diagnosis and its clinical implications can have profound impact if angiographic intervention is feasible, demonstrating clear benefits for multidisciplinary collaboration between physician and interventional radiologist clinicians in diagnosis and management planning.

Balloon-occluded retrograde transvenous obliteration and similar approaches have long been used for treatment of gastric varices following hemorrhage and is being increasingly used for management of HE with large SPSS. Previous studies have used ammonia levels (Mukund et al. 2012), presence/grade of HE, and hospitalization (Laleman et al. 2013)as markers of success. We used two forms of neurocognitive testing to contrast the patient’s ability pre- and post-ARTO. The Number Connection Test is a widely used assessment of visuospatial orientation and psychomotor speed, and is a component of the Psychometric Hepatic Encephalopathy Score (Weissenborn 2015). The patient was shown a sheet of paper with 15 numbered circles randomly spread across the paper and asked to draw a line connecting the circles in order from 1–15. A healthy subject should be able to complete the task within 30 s. Although there was an improvement in our patient from 62 to 55 s, the persistent impairment may reflect minimal HE or another cognitive impairment. The baseline clockface drawing demonstrated constructional apraxia and conceptional deficits (incomplete numbers, inaccurate hand placement) associated with a positive predictive value of 0.96 for HE in patients with cirrhosis (Edwin et al. 2011), with subsequent improvement in visuospatial construction. The reduction in emergency hospital admissions in the three months following ARTO may have multiple contributing factors in addition to an improvement in HE, including reduction in alcohol intake, greater healthcare engagement, and improvement in nutrition. The reduction in emergency hospital admissions in the three months following ARTO may have multiple contributing factors in addition to an improvement in HE, including reduction in alcohol intake, greater healthcare engagement, and improvement in nutrition. Collateral history obtained from the patient’s wife and treating clinicians, suggested that there was a subjective improvement in mood, which is likely interlinked with the other contributing factors. This case demonstrates that both the direct and indirect consequences of accurate identification and treatment of refractory HE impacts not only the patient but their carers and support people.

## Conclusion

ARTO is well-tolerated, safe, and effective for refractory HE in the context of a spontaneous portosystemic shunt. It can be challenging to correlate the presence of such shunts with refractory HE despite the profound impact on quality of life and need for hospitalization. Application of Baveno VI-SPSS criteria to estimate cross-sectional area can assist in identifying shunts likely to contribute to complications including HE and death. Successful obliteration of a culprit SPSS and reduction in HE may be reflected by improvement in neurocognitive testing and reduction in emergency hospitalization.

## Data Availability

Not applicable.

## Abbreviations

ARTO: Angiographic, retrograde transvenous obliteration
CT: Computed tomography
HE: Hepatic encephalopathy
SPSS: Spontaneous portosystemic shunt
